# Protocol for the development of an international Core Outcome Set for treatment trials in adults with epilepsy: the EPilepsy outcome Set for Effectiveness Trials Project (EPSET)

**DOI:** 10.1186/s13063-022-06729-4

**Published:** 2022-11-17

**Authors:** James W. Mitchell, Adam Noble, Gus Baker, Rachel Batchelor, Francesco Brigo, Jakob Christensen, Jacqueline French, Antonio Gil-Nagel, Alla Guekht, Nathalie Jette, Reetta Kälviäinen, John Paul Leach, Melissa Maguire, Terence O’Brien, Felix Rosenow, Philippe Ryvlin, Phil Tittensor, Manjari Tripathi, Eugen Trinka, Samuel Wiebe, Paula R. Williamson, Tony Marson

**Affiliations:** 1 Association of British Neurologists Clinical Research Fellow, Institute of Systems, Molecular and Integrative Biology (ISMIB), University of Liverpool, Liverpool, UK; 2 Health Services Research, Institute of Population Health, Policy and Systems, University of Liverpool, Liverpool, UK; 3 Department of Biostatistics, University of Liverpool, Liverpool, UK; 4 Institute of Systems, Molecular and Integrative Biology (ISMIB), University of Liverpool, Liverpool, UK; 5 University of Liverpool, Liverpool, UK and Secretary General at International Bureau for Epilepsy, Sandyford, Dublin, Ireland; 6 The Oxford Institute of Clinical Psychology Training and Research, University of Oxford, Oxford, UK; 7 Department of Neurology, Hospital of Merano (SABES-ASDAA), Merano-Meran, Italy; 8 Department of Clinical Medicine, Aarhus University, Aarhus, Denmark; 9 NYU Comprehensive Epilepsy Center, New York, USA; 10 Department of Neurology, Hospital Ruber Internacional, Madrid, Spain; 11 Moscow Research and Clinical Center for Neuropsychiatry, Moscow, Russia; 12 Russian National Research Medical University, Moscow, Russia; 13 Department of Neurology, Icahn School of Medicine at Mount Sinai, New York, USA; 14 University of Eastern Finland and Kuopio Epilepsy Center, Kuopio University Hospital, Member of EpiCARE ERN, Kuopio, Finland; 15 School of Medicine, Dentistry & Nursing, University of Glasgow, Glasgow, UK; 16 Leeds Institute of Medical Research, University of Leeds, Leeds, UK; 17 Central Clinical School, Monash University, Melbourne, Australia; 18 Epilepsy Center Frankfurt-Rhine-Main, University Hospital Frankfurt, Goethe-University, Frankfurt, Germany; 19 Department of Clinical Neurosciences, Centre Hospitalier Universitaire Vaudois, Lausanne, Switzerland; 20 The Royal Wolverhampton NHS Trust and Honorary Lecturer, University of Wolverhampton, Wolverhampton, UK; 21 Department of Neurology, Christian Doppler University Hospital, Paracelsus Medical University Salzburg, Salzburg, Austria; 22 Department of Neurology, Neurosciences Centre, All India Institute of Medical Sciences, New Delhi, India; 23 Department of Clinical Neurosciences, Cumming School of Medicine, University of Calgary, Calgary, Canada

**Keywords:** Core outcome set, Epilepsy, Consensus, Delphi study, Treatment outcome, Clinical trials

## Abstract

**Background:**

A Core Outcome Set (COS) is a standardised list of outcomes that should be reported as a minimum in all clinical trials. In epilepsy, the choice of outcomes varies widely among existing studies, particularly in clinical trials. This diminishes opportunities for informed decision-making, contributes to research waste and is a barrier to integrating findings in systematic reviews and meta-analyses. Furthermore, the outcomes currently being measured may not reflect what is important to people with epilepsy.

Therefore, we aim to develop a COS specific to clinical effectiveness research for adults with epilepsy using Delphi consensus methodology.

**Methods:**

The EPSET Study will comprise of three phases and follow the core methodological principles as outlined by the Core Outcome Measures in Effectiveness Trials (COMET) Initiative. Phase 1 will include two focused literature reviews to identify candidate outcomes from the qualitative literature and current outcome measurement practice in phase III and phase IV clinical trials. Phase 2 aims to achieve international consensus to define which outcomes should be measured as a minimum in future trials, using a Delphi process including an online consensus meeting involving key stakeholders. Phase 3 will involve dissemination of the ratified COS to facilitate uptake in future trials and the planning of further research to identify the most appropriate measurement instruments to use to capture the COS in research practice.

**Discussion:**

Harmonising outcome measurement across future clinical trials should ensure that the outcomes measured are relevant to patients and health services, and allow for more meaningful results to be obtained.

**Core Outcome Set registration:**

COMET Initiative as study 118.

## Background

Randomised controlled trials (RCTs) are the gold standard source of evidence informing treatment decisions for people with epilepsy (PWE). RCTs evaluate the effect of an intervention on outcomes, which should be predefined by the research team.

In epilepsy, the choice of outcome measures varies widely among studies [1] and may not reflect what is important to PWE. This diminishes opportunities for informed decision-making, contributes to research waste and is a barrier to integrating findings from multiple RCTs in systematic reviews and meta-analyses.

For other chronic conditions, there has been an increasing international effort to identify Core Outcome Sets (COS), deriving consensus among people affected by that condition and other relevant key stakeholders (patient representatives, clinicians, and clinical researchers) as to which outcomes should be reported as a minimum [2–4]. COS facilitate the undertaking of trials that are relevant to patients and health services and help standardise trial methodology. They allow for research that is representative and applicable globally, and the standardisation of outcome measurement means that more meaningful results can be obtained from systematic review and meta-analysis [5]. The importance of the use of COS is increasingly recognised by research funders, for instance, the National Institute for Health Research’s Health Technology Assessment programme in the UK and the Health Research Board in Ireland both encouraging COS use in funding applications for new studies. They are also recommended by Trial Registries, including the ISRCTN registry, a primary clinical trial registry recognised by the World Health Organization (WHO) and International Committee of Medical Journal Editors (ICMJE) [6] and regulators including the European Medicines Agency (EMA) [7].

### Aim

To develop a COS for use in treatment trials for adults with epilepsy by using the Delphi consensus methodology and an international consensus meeting.

### Scope

This COS is being developed for:

- Adults (≥18 years) diagnosed with epilepsy. This is to include *all* subtypes of epilepsy and seizures as defined in 2017 by the ILAE Commission for Classification and Terminology [8, 9].
- All non-surgical therapeutic interventions. This includes pharmaceutical, behavioural, psychological and complex interventions.
- For use in clinical effectiveness research investigating treatment for adults with epilepsy.

Surgical interventions including neuromodulation are outside of the scope of this COS. Whilst many desirable outcomes are anticipated to overlap when considering surgical and non-surgical treatment, it is anticipated that some core outcomes related to adverse events may differ.

Outcomes relating to the broad domains of physiological outcomes, clinical outcomes, life impact outcomes, resource use outcomes and adverse events will be considered.

## Methods/design

**Fig. 1:**
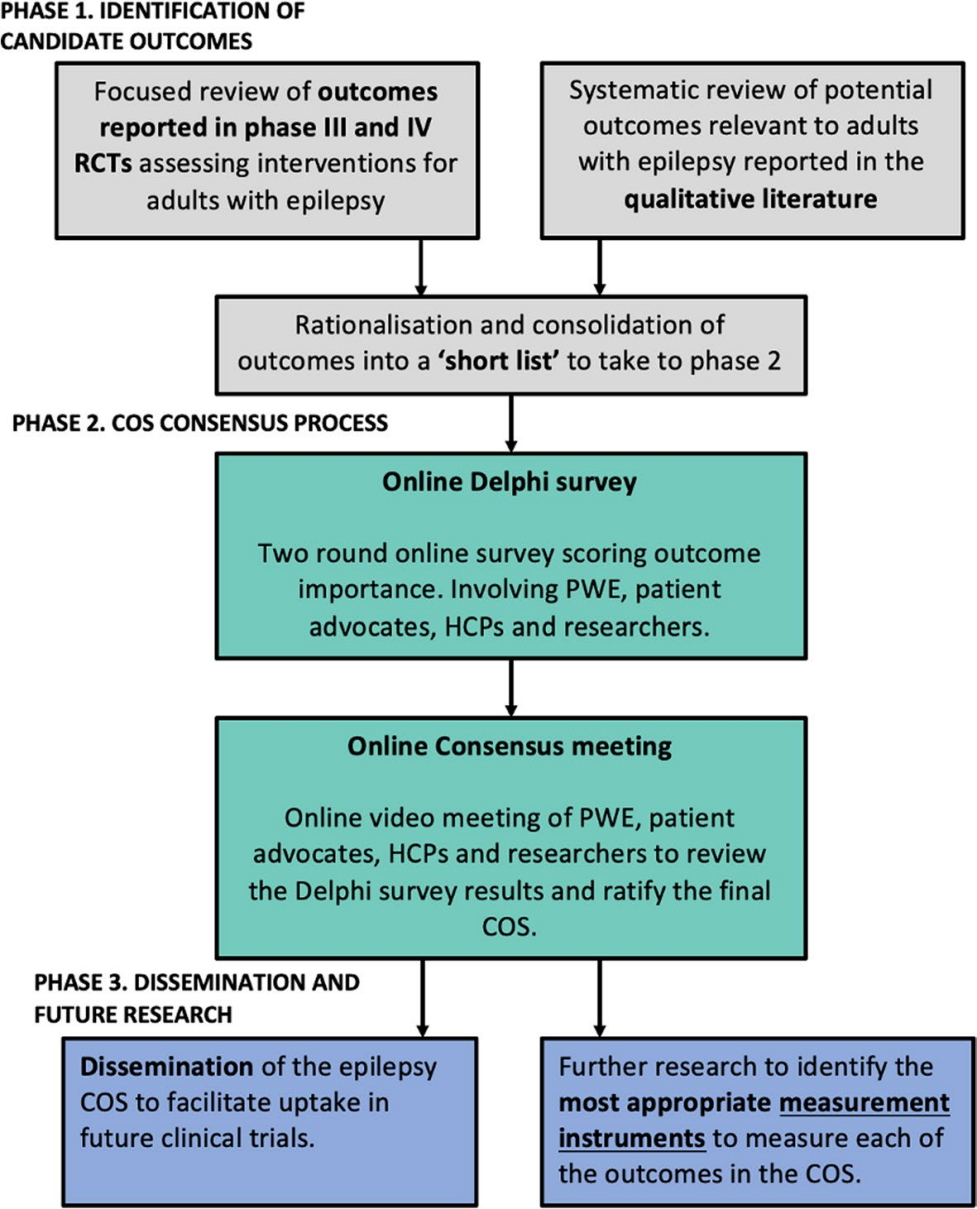
EPSET Project study flowchart. Abbreviations: RCT, randomised controlled trial; COS, Core Outcome Set; PWE, people with epilepsy; HCP, healthcare professional

The EPSET Study will comprise of three phases (see Fig. 1) and follow the core methodological principles as outlined by the Core Outcome Measures in Effectiveness Trials (COMET) Initiative. Phase 1 will comprise of focused literature reviews to identify candidate outcomes from the qualitative literature and current outcome measurement practice in adult epilepsy phase III and phase IV randomised controlled trials (RCTs). Phase 2 aims to achieve international consensus to define which outcomes should be measured as a minimum in future trials, using a Delphi process including an online consensus meeting involving key stakeholders. Phase 3 will involve dissemination of the ratified COS to facilitate uptake in future trials and the planning of further research to identify the most appropriate measurement instruments to use to capture the COS in research practice.

Core Outcome Set standards for development, the COS-STAD recommendations, will be followed at all stages of this study [10].

### PHASE 1 – identification of candidate outcomes

The aim of Phase 1 is to generate a comprehensive list of candidate outcomes relating to the treatment of epilepsy in adults. This list will be generated by extracting potential outcomes from the published qualitative literature exploring ‘what is important’ to adults with epilepsy (Review A), and extracting outcomes already measured in clinical trials investigating treatments for adults with epilepsy (Review B).

#### Review A – qualitative literature review

This focused systematic review will identify potential outcomes identified as important to adults with epilepsy and their representatives (including family and caregivers) from the published qualitative literature. We will search the single health-related database MEDLINE using an established qualitative methodological filter that demonstrates high sensitivity and precision to identify qualitative literature [11]. Studies reporting qualitative primary evidence of the views and experiences of adults with epilepsy and their caregivers, written in English with no date restrictions will be eligible for inclusion. Abstract and full-text review to determine article eligibility will be performed by two independent researchers. Potential outcomes will be coded from the verbatim participant data and mapped to the COMET taxonomy of outcomes used by COS developers to classify outcome types [12]. This taxonomy is well established in COS development and provides a standardised high-level classification system that facilitates uniformity of outcome classification across electronic databases.

The full protocol including the study selection criteria is available online via the PROSPERO database [13].

#### Review B – review of outcomes from clinical rrials

This review will identify which outcomes are currently being measured in epilepsy research assessing the effectiveness of interventions for adults with epilepsy.

We will identify via trial registry entries, consecutive phase III and IV epilepsy treatment randomised controlled trials (RCTs) assessing non-surgical treatments for adults with epilepsy. RCTs registered on clinicaltrials.gov and ISRCTN registry databases from 1st January 2000 to 1st January 2022 will be reviewed. Results publications will be eligible if they include the clinicaltrials.gov identifier within the body of the text or are published within the clinicaltrials.gov database. Validation by a second researcher checking 10% of the database entries will be performed to ensure the quality of study selection. Outcomes including measurement instruments, where available, will be extracted verbatim in the most granular form to ensure that the meaning of outcomes is not misrepresented.

#### Consolidation of outcomes into a ‘short list’

The candidate verbatim outcome terms from Review A and Review B will be categorised in line with the COMET outcome domain taxonomy to aid the conceptualisation of the outcomes [12]. This list will be rationalised and into a feasible number of summary outcomes to take forward to the consensus process. This rationalisation will be performed and overseen by the study management team (JWM, AN, PW, TM) and a patient representative to ensure that the outcomes in the ‘short list’ are representative of the more granular outcomes. An example of this consolidation process for the identification of one summary candidate outcome is outlined in Table 1.

**Table 1:** Example of consolidation of granular outcomes from Phase 1 into a summary candidate outcome

Granular verbatim outcome from Reviews A and B	Summary candidate outcome (descriptor)
1. ‘hospital admission’	HOSPITAL ATTENDANCE OR ADMISSION Definition - the need to attend hospital or the emergency department due to epilepsy, excluding routine clinic appointments
2. ‘emergency department attendance’	
3. ‘avoidable hospital attendance’	
4. ‘number of hospital admissions due to epilepsy over [predefined period]’	

It is important to consolidate the outcome list, given that evidence from previous COS developers has identified that a higher number of items to score in the consensus process is associated with significantly lower survey response rates and participant attrition [14].

### PHASE 2 – COS consensus process

The summary candidate outcomes will be taken to an international, multistakeholder consensus process involving a two-round, online Delphi survey followed by an online consensus meeting, to decide which outcomes should be prioritised and classified as ‘core outcomes’. Before this, the outcomes and associated description text will be pilot tested with a small group of people with epilepsy identified from epilepsy patient organisations to ensure optimum clarity, readability, and acceptability of the survey format. Outcomes will be presented to stakeholders organised in line with the COMET taxonomy of outcomes [12].

#### Stakeholder groups

The following stakeholder groups from around the world will be invited to participate in both the Delphi surveys and the online consensus meeting:

1. *Adults with epilepsy* aged 18 years and older able to complete the Delphi survey.
2. *Patient representatives including family members and/or caregivers* of adults with epilepsy where an adult with epilepsy is unable to provide online consent and/or complete the Delphi survey.
3. *Healthcare professionals* who regularly assess and treat adults with epilepsy (neurologists, epileptologists, epilepsy specialist nurses and allied healthcare practitioners)
4. *Researchers* involved in assessing interventions for adults with epilepsy, particularly clinical trialists.

Stakeholders will be identified and invited using communication channels through professional organisations such as the International League Against Epilepsy (ILAE) and its regional chapters, patient charities and advocacy groups as well as professional contacts of the international working group.

To ensure that the COS is globally representative, participants will be invited to participate in English and several other languages selected by the international working group, where the inclusion of a translation is anticipated to improve stakeholder participation from a region. Translation will occur using a forward and bilingual backwards translation process, with the backwards translated versions of the Delphi survey checked for inconsistencies by the Study Management Team prior to distribution. Given the nature of the consensus meeting, it will not be possible for participants who are unable to contribute in English to participate. Despite this, we will be including views of non-English speakers in the consensus process by involvement in the Delphi surveys.

#### Online Delphi survey

The list of candidate outcomes will be voted on in the two-round online Delphi Survey, delivered using the DelphiManager software, designed and hosted by the University of Liverpool, UK [15]. The Delphi method will be used, which allows for participants to consecutively score the importance of outcomes in multiple rounds, as a means of obtaining consensus. The Delphi method allows anonymous review and scoring of outcomes in a way that gives equal influence to all who participate, avoids an individual participant being overtly influenced by the opinions of any other participant, facilitates international contribution and provides a mechanism for reconciling different opinions [16].

In the first round, the piloted candidate outcomes and associated description text will be presented. The order in which the outcomes are presented to participants will be randomly generated for each participant, to remove the likelihood of question order bias influencing the results. Scoring of outcomes will use the Grading of Recommendations, Assessment, Development and Evaluations (GRADE) nine point Likert scale [17]. Scoring options will be labelled as 1 to 3 ‘not that important’, 4 to 6 ‘important but not critical’ and 7 to 9 ‘critical’. An ‘unable to score’ option will also be available for participants to use. To identify potentially important domains not on the list, participants will be able to use a free text box to suggest outcomes which they think are important but not already included. Novel outcomes identified at this stage will be reviewed by the entire study management team and a patient representative to decide whether they meet the scope of the research, should be included in the second round of the survey, or whether existing outcome descriptions should be modified.

In the second round, each participant will be presented with the same candidate outcomes they scored in round one. Before scoring them again, participants will be presented with their previous scores as well as a graphical summary of the round one results for all participants that completed the survey, displayed separately for each stakeholder group. After comparing the scores across the stakeholder groups, they will be able to score each outcome a second time. Participants may choose to change their score or keep it the same. For any novel outcomes introduced in the second round, participants will be asked to score these for the first time.

Participants will be encouraged to provide a score for each outcome. Responses will be included in the analysis if a participant scores 70% or more of the outcomes; otherwise, the results from that participant will be treated as incomplete and excluded from the analysis.

Once the results from the second round of the Delphi survey have been summarised, each outcome will be classified as to whether it has met consensus using a priori criteria (Table 2).

**Table 2:** EPSET study definition of consensus for the Core Outcome Set (COS)

Classification	Description	Criterion
Consensus ‘in’	Consensus that the outcome should be included in the COS	80% or more participants scoring 7 to 9 AND less than 10% scoring 1-3 in all stakeholder groups
Consensus ‘out’	Consensus that the outcome should not be included in the COS	50% or less participants scoring 7 to 9 in all stakeholder groups
No consensus	Uncertainty about the important of the outcome. Further discussion is required at the consensus meeting	Any other scoring

The 80/10% rule for consensus ‘in’ will be used, as the situation where 80% or more of participants score an outcome as 7 to 9 on the Likert scale with 10% or less scoring it 1 to 3 represents a scenario where the majority believe that the item is critical to include in the core outcome set and only a small minority think it is of little or no importance. Defining such consensus criteria is important because using a threshold that is too accommodating risks generating a long list of outcomes that may be challenging to implement in practice, whereas using criteria that are too stringent risks excluding outcomes that a large proportion of participants feel are essential.

#### Reducing attrition

To reduce the number of participants not completing the surveys, reminder emails to registered participants who have not yet completed the survey will be sent (up to a maximum of 3 reminder emails for each round), in addition to social media promotion.

#### Participant invitation and consent process

People with epilepsy and their representatives will be invited to take part by the following routes:

- Invitation email to participants distributed by epilepsy charities, and patient advocacy groups. This process will be guided by local data protection and privacy laws as well as individual organisational policies on member communications.
- Promotion with a link to the online survey from social media platforms and websites of epilepsy charities and patient advocacy groups.

Healthcare professionals and researchers will be invited to take part by the following routes:

- Invitation email to participants distributed by professional epilepsy organisations (e.g. The ILAE), epilepsy charities and personal email distribution lists of the study team.
- Invitation during engagement at conferences.
- Promotion with a link to the online survey from social media platforms and websites of professional organisations.
- Promotion via professional newsletters and publications.

Consent from participants in the online survey will be sought online prior to accessing the survey, along with screening questions to confirm eligibility. Participants will be reminded that they are free to withdraw from the survey at any time without giving a reason, and can request that their survey responses are withdrawn from analysis, if not already presented. There is no restriction on the number of eligible participants.

#### The consensus meeting

The results of the second round Delphi survey will be discussed in an online video consensus meeting using a password-protected video-conferencing platform. This meeting will be chaired by an independent facilitator, without experience of living with or treating people with epilepsy, but with an understanding of clinical trial methodology to reduce the risk of bias in the facilitation process.

Participants will be invited if they have indicated at the end of the second round of the Delphi survey that they would like to take part. Attempts will be made to have an equal number of participants from each stakeholder group in the consensus meeting. If more than 50 participants chose to take part in the online consensus meeting, then participants will be selected purposively to represent the broadest range of views and experience from around the world. In addition, a small number of members of regulatory agencies (e.g. the FDA and EMA) and funding bodies (e.g. National Institute of Neurological Disorders and Stroke in the USA) will be invited to the meeting as non-voting participants. This is to improve engagement with the COS development process and improve uptake of the COS in research practice once published. Attendance at the meeting will be taken as consent to participate.

Each of the outcomes which have not met consensus from the second round Delphi survey will be discussed in detail. Following this discussion, repeat scoring to decide on whether to include an item in the Core Outcome Set will take place. Consensus to include an item will be defined as 80% or more of each stakeholder group, as previously defined, voting as critical (7–9 on the same 9-point Likert scale used in the Delphi process).

An online consensus meeting is being used as opposed to a face-to-face meeting as this method facilitates wider geographical representation, involvement of more diverse stakeholders and also reduces the environmental impact of international travel [18, 19].

#### Data analysis plan

Demographic characteristics including participant age, gender, country of residence, and survey language option chosen will be analysed using descriptive statistics, and reported for the whole group and by each stakeholder group. Data on employment status, years since epilepsy diagnosis, average seizure frequency and self-reported hospital utilisation due to epilepsy will be collected from patient participants, and also analysed using descriptive statistics.

Outcome voting response options will be presented as the proportion of participants voting an outcome as 1 to 3 ‘not that important’, 4 to 6 ‘important but not critical’, 7 to 9 ‘critical’ and ‘unable to score’ for all participants, and by stakeholder group, for each outcome. Graphical representations of these responses will be presented to participants between Delphi survey rounds, and in the final manuscript as a supplementary document.

Comparative analysis will not be performed, as this is not required for Delphi consensus methods.

### PHASE 3 – dissemination of the COS and future research

The final Core Outcome Set (COS) represents ‘what’ outcomes should be measured in future research. This will be disseminated by presentation at international epilepsy and research methodology conferences and published in a peer-review journal. The COS will also be publicised to regulatory bodies (e.g. the EMA and FDA), clinical trial registries, funding bodies and the pharmaceutical industry to encourage uptake of the COS in future studies.

Often researchers use different measurement instruments to measure the same construct, and it can therefore be difficult to compare and contrast results from different studies, further contributing to research waste. Therefore, the dissemination process will also seek to develop plans and support for an international project to establish consensus on ‘how’ the different outcomes included in the COS should be measured (i.e. which instruments should be used).

## Discussion

This study protocol presents the methodology for the development of a COS for clinical effectiveness research involving adults with epilepsy in line with Core Outcome Measures in Effectiveness Trials (COMET) Initiative recommendations [20].

Developing the COS is the first step in improving the measurement of outcomes in clinical trials for adults with epilepsy. Once the COS is defined, further research will be needed to determine the measurement methods or measurement instruments available for each of the core outcomes, followed by an assessment of the quality of and feasibility of using these methods. This will allow for a recommendation of the most appropriate measurement methods to capture the COS. This process will also require the input of key stakeholders at all stages of the research process, including people with epilepsy, their advocates, clinicians, clinical researchers, and experts in measurement instrument development and analysis.

## Registration

This study has been registered with the COMET Initiative as study 118 (http://www.comet-initiative.org/studies/details/118) and will follow its recommended methodological procedures [20].

## Study status

Phase 1 of the study is in progress and commenced in November 2020. Phase 2 is expected to commence in Spring 2022 and the COS ratified by Autumn 2022. Recruitment to the Delphi process had not started at the time of manuscript submission.

## Data Availability

The final dataset from the Delphi process will be accessible for analysis by the Study Management Team (JWM, AN, PW, TM). Summary data presented with response per question organised by stakeholder group will be available to the entire Working Group throughout the data analysis phase and also submitted as an appendix with the results journal publication.

## Abbreviations

COMET: Core Outcome Measures in Effectiveness Trials
COS: Core Outcome Set
EMA: European Medicines Agency
FDA: Food and Drug Administration
ICMJE: International Committee of Medical Journal Editors
ISRCTN: International Standard Randomised Controlled Trial Number
PWE: People with epilepsy
RCT: Randomised controlled trial
WHO: World Health Organization
